# A 5-year review of Helicobacter pylori isolated from gastric biopsies at the UK Health Security Agency Gastrointestinal Bacteria Reference Unit

**DOI:** 10.1099/jmm.0.002142

**Published:** 2026-03-18

**Authors:** Craig Swift, Claire Jenkins, Marie Anne Chattaway, Vanessa Wong, Derren Ready, Gauri Godbole

**Affiliations:** 1 UK Health Security Agency, London, England, UK; 2 NIHR Health Protection Research Unit, Gastrointestinal Infections, University of East Anglia, Norwich, UK; 3 NIHR Health Protection Research Unit, Evaluation and Behavioural Science, University of Bristol, Bristol, UK

**Keywords:** antimicrobial, biopsy, gastric, *Helicobacter pylori*, resistance, UK

## Abstract

**Introduction.** *Helicobacter pylori* colonizes large proportions of the global population and is associated with the development of gastric ulcers and cancer. Antimicrobial resistance to multiple first-line antibiotics is a major contributing factor in treatment failure.

**Gap statement.** Within the UK, there is limited up-to-date information on antimicrobial susceptibility and resistance (AMR) rates in *H. pylori*.

**Aim.** To assess increasing gastric biopsy referrals and temporal trends in antimicrobial resistance in *H. pylori* recovered from gastric biopsy specimens by the Gastrointestinal Bacteria Reference Unit (GBRU) over a 5-year period (2020 to 2024).

**Methodology.** *H. pylori* was recovered from gastric biopsies of patients predominantly with recurrent/persistent infection, collected by endoscopy across the UK through culture on selective and non-selective agar media. Antimicrobial susceptibility to metronidazole, clarithromycin, levofloxacin, amoxicillin and tetracycline was determined phenotypically by E-test.

**Results.** Twelve thousand six hundred sixty gastric biopsy specimens were received from which viable *H. pylori* was successfully recovered and susceptibility testing was performed for 40.5%, with 53.9% recovered from specimens received within 1 day following collection and decreasing to <35.0% after 4 days. Most (96.1%) were found to be resistant to metronidazole, followed by clarithromycin (82.2%) and levofloxacin (31.5%), with resistance to amoxicillin (1.2%) and tetracycline (0.2%) rarely detected.

**Conclusion.** Within treatment-refractory patients in the UK, there is a high prevalence of resistance to metronidazole and clarithromycin. There is an urgent need to establish UK primary resistance rates in treatment-naïve patients to support a review of regional first-line treatment regimes. These results support a need for rapid, local isolation of *H. pylori* by clinical microbiology laboratories to maximize patient benefit due to delays in testing, significantly reducing the likelihood of recovering viable strains for susceptibility testing, and demonstrate that implementation of molecular tests could support better patient management.

## Introduction

*Helicobacter pylori* is a Gram-negative, flagellated, helical bacterium that can colonize the mucous lining of the stomach; it is present in a large proportion of individuals globally. Although colonization will not always cause illness, persistent colonisation with virulent strains may lead to clinical conditions including chronic gastritis, chronic dyspepsia and gastric and duodenal peptic ulcer disease. Furthermore, *H. pylori* is a biological carcinogen and a primary risk factor for gastric cancer and gastric mucosa-associated lymphoid tissue lymphoma [1,2]. Diagnostic approaches for *H. pylori* include non-invasive tests such as serology, stool antigen detection and the urea breath test, as well as invasive methods like endoscopic biopsy.

Treatment for infections requires a minimum of two antimicrobial agents in combination with a proton pump inhibitor. As few agents are effective in the gastric niche, only four antimicrobials, specifically metronidazole, clarithromycin, tetracycline and amoxicillin, are recommended in the UK guidelines for eradication regimens [3]. Antimicrobial resistance, particularly to clarithromycin and metronidazole, is a major contributing factor in treatment failure [4]. In the UK, alternative ‘rescue’ therapies are available, with these incorporating antimicrobial agents not used in first- or second-line therapies, including (unlicensed) levofloxacin and tetracycline. Despite the recent decision to remove clarithromycin-resistant *H. pylori* from the WHO Bacterial Priority Pathogens List 2024 [5], globally, the management of *H. pylori* infection remains challenging due to the prevalence of antimicrobial resistance.

In the UK, national guidelines recommend collection of gastric biopsies for *H. pylori* culture and antimicrobial susceptibility testing, to confirm infection and to inform appropriate treatment strategies for refractory patients with dyspepsia who have failed first- and second-line treatments and those who have limited options due to drug hypersensitivity or live in an area with high resistance rates [6]. However, *H. pylori* recovery from biopsy samples may be compromised by strain fastidiousness, effects of therapy causing low cell density and loss of viability and/or overgrowth of contaminating microorganisms resulting from delays introduced whilst transporting specimens.

The aim of this study was to review microbiological data, including isolation rates and susceptibility profiles, from gastric biopsies referred to the UKHSA Gastrointestinal Bacteria Reference Unit (GBRU) over a 5-year period (2020–2024).

## Methods

### Culture and isolation

All gastric biopsies referred to the GBRU from 1 January 2020 to 31 December 2024 were included in this study. *H. pylori* was cultured on 10% Columbia blood agar (UKHSA media services) and *H. pylori* selective media (E & O Laboratories Ltd). Plates were incubated at 35–38 °C under microaerophilic conditions (4% O_2_, 5% CO_2_, 86% N_2_ and 5% H_2_) in a M45 Microaerophilic Workstation (Don Whitley Scientific, Shipley, UK) for up to 10 days. Identification of *H. pylori* was confirmed by Gram-staining, catalase, oxidase and urease reactions or the Allplex™ *H. pylori* and ClariR Assay (Seegene Inc).

### Phenotypic antimicrobial susceptibility testing

Cultures of *H. pylori* were tested alongside the control strain NTCC12455 for phenotypic susceptibility to five antimicrobial agents (amoxicillin, clarithromycin, levofloxacin, metronidazole and tetracycline) using gradient strips E-tests (BioMérieux). Inoculum was adjusted to McFarland 3 and spread onto the surface of Mueller–Hinton agar with the inclusion of 10% blood before gradient strips were applied. Plates were immediately incubated between 4 and 5 days under microaerophilic conditions (4% O_2_, 5% CO_2_, 86% N_2_ and 5% H_2_) in a M45 Microaerophilic Workstation (Don Whitley Scientific). The MICs were interpreted using the European Committee on Antimicrobial Susceptibility Testing (EUCAST) *H. pylori* clinical breakpoints relevant for each year (EUCAST Clinical Breakpoint Tables: v10.0 for 2020 through v14.0 for 2024).

## Results

**Fig. 1.**
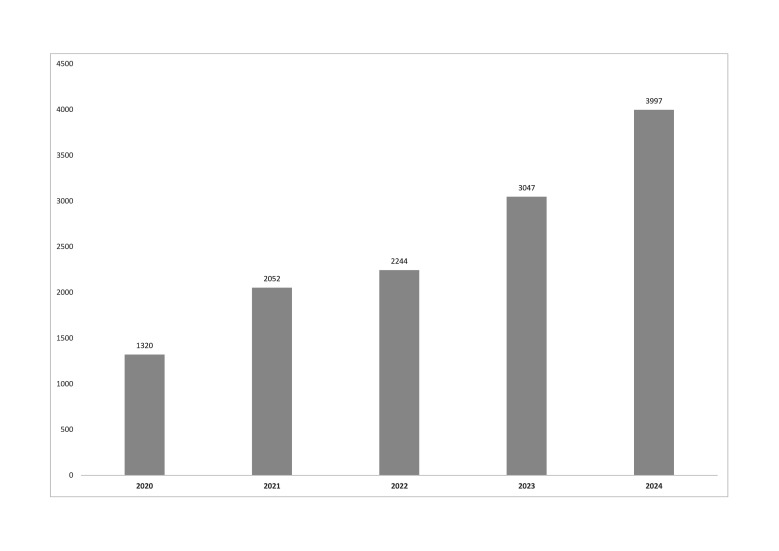
Annual number of gastric biopsy specimens received by the GBRU for the isolation of *H. pylori*.

Between January 2020 and December 2024, a total of 12,660 gastric biopsies were received by the GBRU for the isolation of * H. pylori* (Fig. 1), with annual sample numbers almost doubling from 2021 to 2024, likely due to ongoing post-Coronavirus disease 2019 (COVID-19) recovery of clinical services within NHS laboratories.

**Fig. 2.**
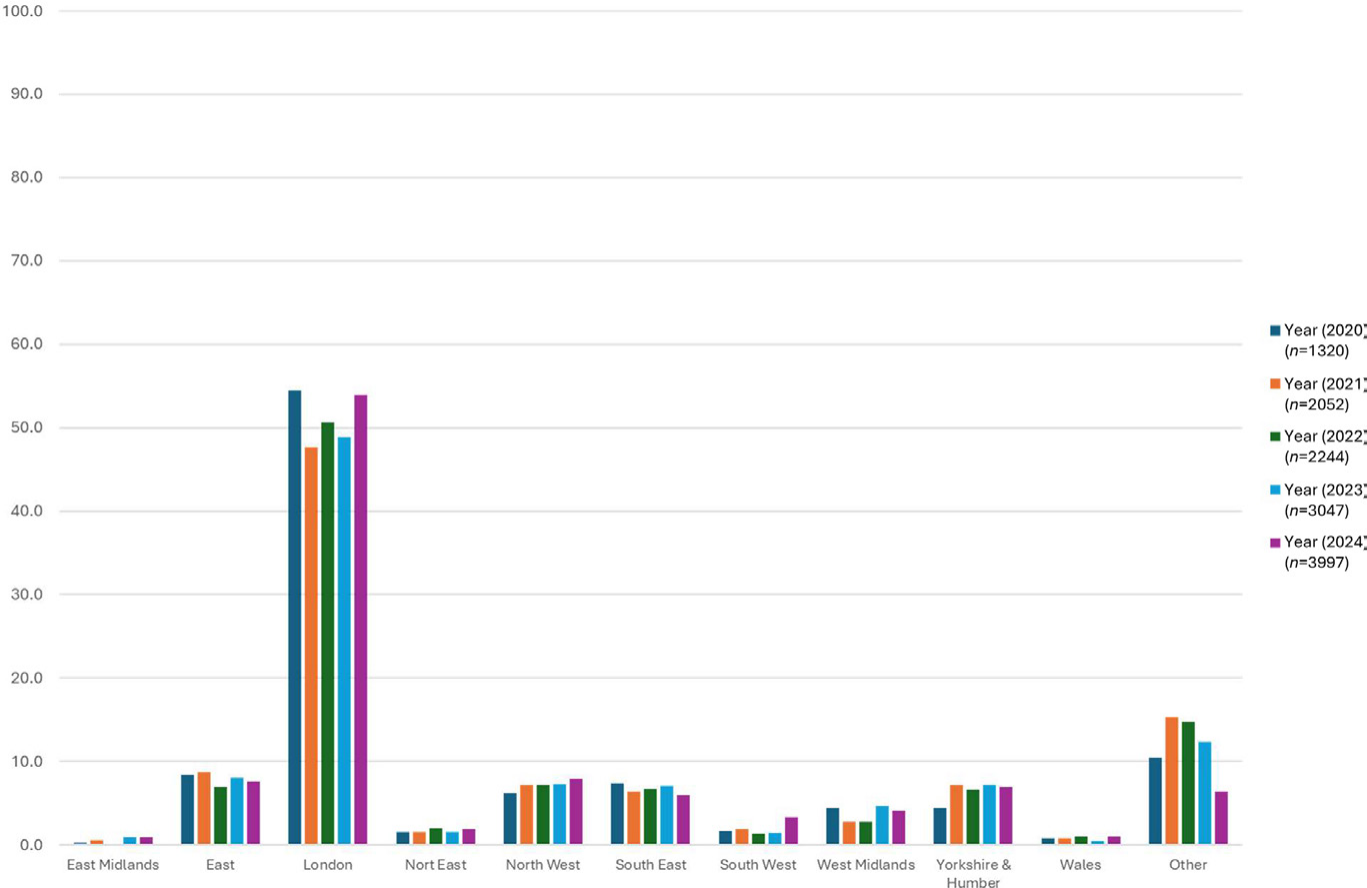
Annual regional proportions of gastric biopsies received by the GBRU for the isolation of *H. pylori*.

Just over half of the specimens, 6,447/12,660 (51.2%) received during this timeframe were referred from laboratories in the London region, while the other 8 regions within England (East Midlands, East, North East, North West, South East, South West, West Midlands and Yorkshire and Humber) were represented by <8.0% each, with East Midlands referring the fewest number of specimens (Fig. 2).

**Fig. 3.**
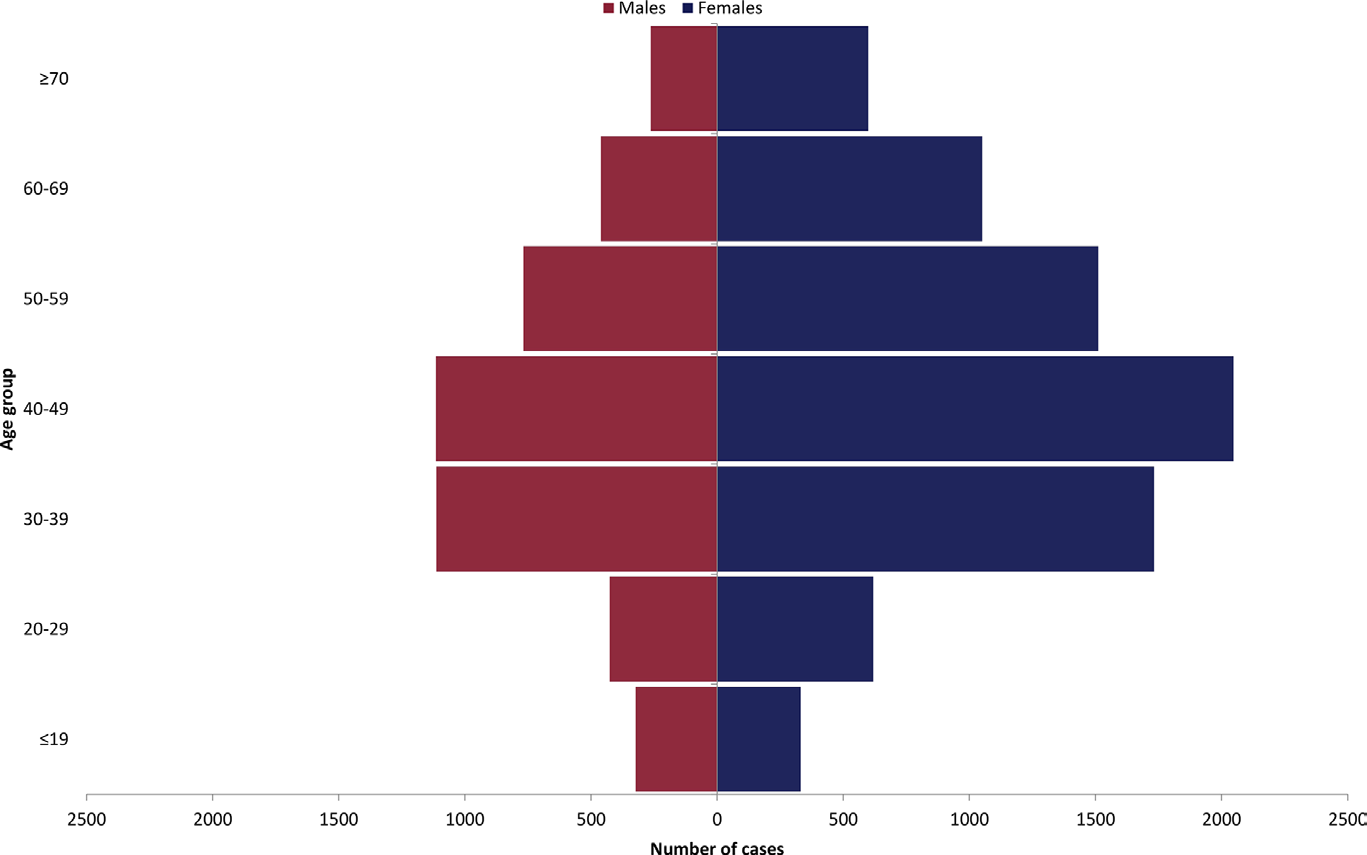
Age and sex demographic of gastric biopsy specimens received by the GBRU for the isolation of *H. pylori* over a 5-year period (2020–2024).

Age and sex demographics were available for 12,359/12,660 (97.6%) (Fig. 3). The overall pattern shows higher representation of females across most age groups, with predominance in the 40–49 age group.

**Fig. 4.**
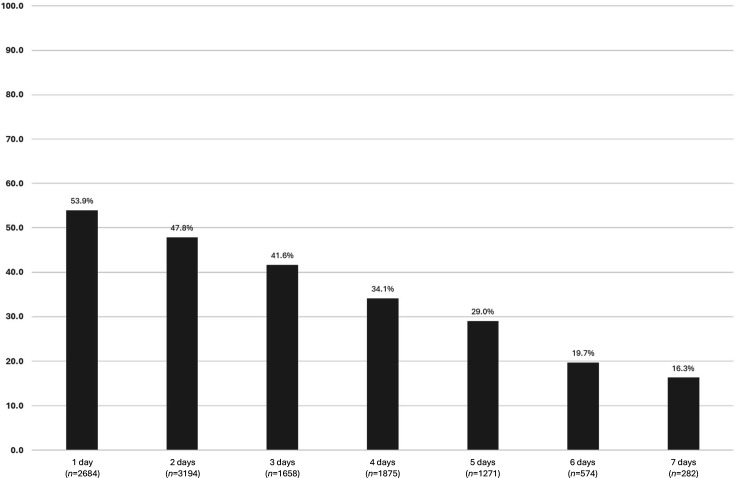
Proportion of gastric biopsy specimens from which *H. pylori* was successfully recovered (*n*=11,538).

The proportion of culture-positive biopsies remained relatively stable, ranging from 36 to 44% over the 5-year period. Collection dates were available for 11,538/12,660 (91.1%) specimens. Amongst these, successful recovery of *H. pylori* was correlated in relation to the number of days taken from specimen collection to receipt for testing by the GBRU. The proportion of biopsies which were culture positive for *H. pylori* progressively declined in relation to increasing time from sample collection, from >50.0% at day 1 to <20.0% at days 6–7 (Fig. 4).

### Antimicrobial susceptibility testing

**Fig. 5.**
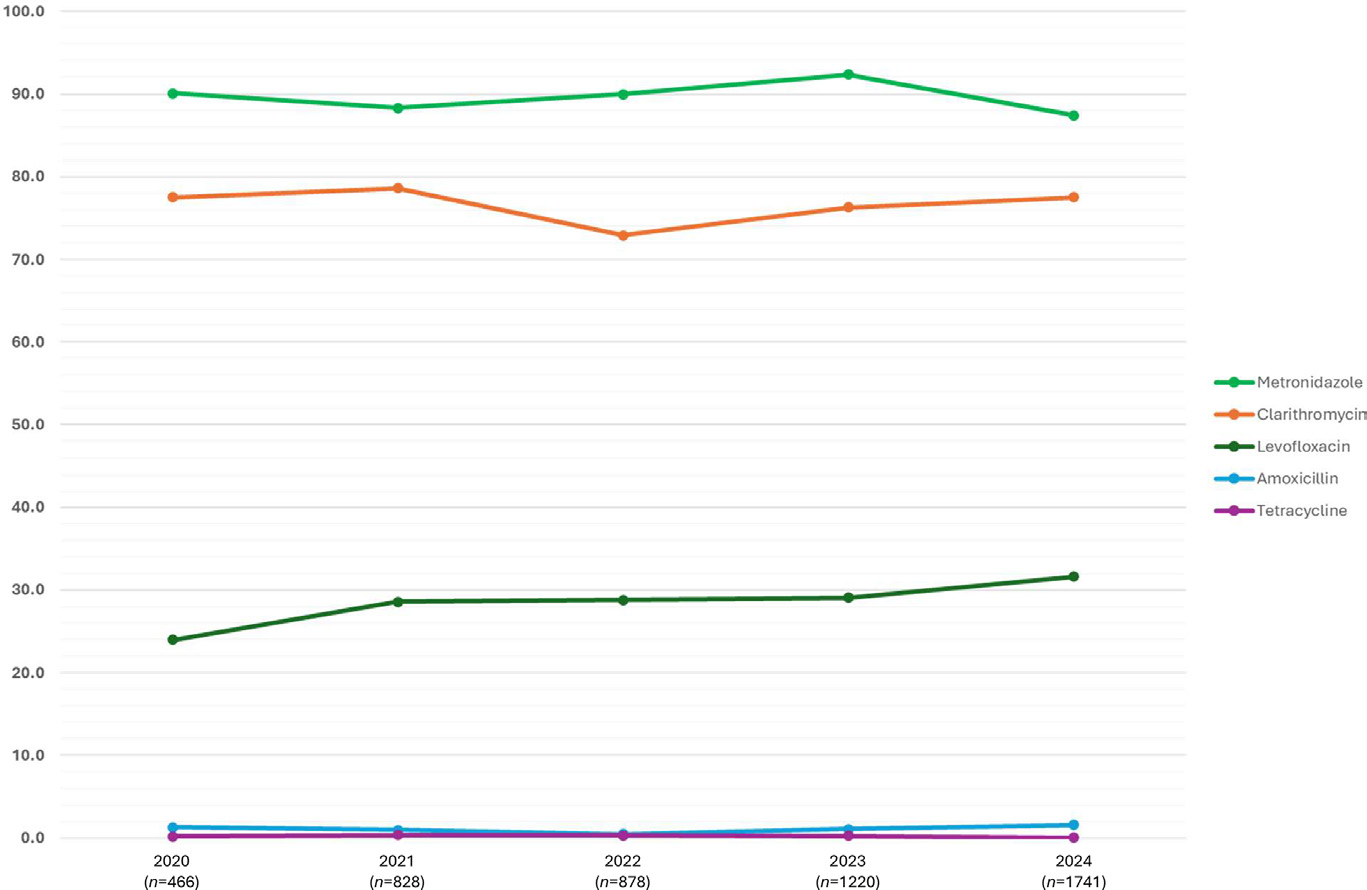
Proportions of phenotypic antimicrobial resistance to the five key antimicrobials tested by the GBRU (*n*=5,133).

Antimicrobial susceptibility testing was performed on 5,133 cultures of *H. pylori* successfully recovered from gastric biopsies referred to the GBRU between 2020 and 2024. Most (4,594/5,133; 96.1%) were found to be resistant to metronidazole, followed by clarithromycin (3,932/5,133; 82.2%) and levofloxacin (1,507/5,133, 31.5%), with resistance to amoxicillin (59/5,133; 1.2%) and tetracycline (11/5,133; 0.2%) rarely detected. The prevalence of resistance to levofloxacin increased from 24.0% in 2020 to 31.6% in 2024 (Fig. 5, Table 1).

**Table 1.** Number and proportion of *H. pylori* cultures exhibiting resistance to the five key antimicrobials tested by the GBRU (2020–2024)

Antimicrobial agent (% of resistant cultures)	Year (*n*=total no. of cultures)				
	2020 (*n*=466)	2021 (*n*=828)	2022 (*n*=878)	2023 (*n*=877)	2024 (*n*=1,741)
Metronidazole	420 (90.1%)	731 (88.3%)	790 (90.0%)	1,127 (92.4%)	1,526 (87.7%)
Clarithromycin	361 (77.5%)	651 (78.6%)	640 (72.9%)	931 (76.3%)	1,349 (77.5%)
Levofloxacin	112 (24.0%)	237 (28.6%)	253 (28.8%)	355 (29.1%)	550 (31.6%)
Amoxicillin	6 (1.3%)	8 (1.0%)	4 (0.5%)	13 (1.1%)	28 (1.6%)
Tetracycline	1 (0.2%)	3 (0.4%)	3 (0.3%)	3 (0.2%)	1 (0.1%)

There were 3,615/5,133 (70.4%) cultures resistant to both metronidazole and clarithromycin, 1,234/5,133 (24.0%) were resistant to metronidazole, clarithromycin and levofloxacin and 36/5,133 (0.7%) were resistant to metronidazole, clarithromycin, levofloxacin and amoxicillin. No cultures were found to be resistant to all five antimicrobial agents tested, and 202/5,133 (3.9%) were susceptible to all five.

## Discussion

The management of *H. pylori* infection is becoming a global challenge due to increasing antimicrobial resistance. Infection with this pathogen can lead to conditions such as chronic gastritis and gastric ulcers, as well as increase the risk of developing gastric cancer. According to the international guidelines, Maastricht VI/Florence Consensus [7], the first-line eradication therapy should be based on the prevalence of local clarithromycin-resistant *H. pylori* strains. If the clarithromycin resistance rate is equal to or more than 15% of all diagnosed *H. pylori* infections in a population, eradication treatment with a clarithromycin-containing protocol is not recommended without prior susceptibility testing [7,8]. However, a lack of *H. pylori* susceptibility testing in treatment-naive patients in most local and regional microbiology laboratories in England is responsible for a knowledge gap that hinders effective susceptibility-based antimicrobial therapy that can contribute to an increasing prevalence of treatment failure [7].

While most specimens received for testing each year were from laboratories in the London region, which may reflect a greater awareness of the services provided by the GBRU, this review highlights an overall increasing trend of gastric biopsy referrals to our laboratory over the duration of the last 5 years (2020–2025). Further analysis is required to determine if this is due to ongoing post-COVID-19 recovery of clinical services within NHS laboratories or linked to an emerging trend of failed treatments. Nevertheless, there remains an acute need for surveillance of primary antimicrobial susceptibility and resistance (AMR) in * H. pylori* within England to enable guided antimicrobial treatment for eradication.

In *H. pylori* recovered from gastric biopsy specimens over this 5-year period, there is a clear correlation between the ability to successfully recover *H. pylori* from gastric biopsy specimens by culture in our laboratory and increasing time delay between collection and receipt, clearly highlighting the limitation of culture-based testing for specimens received more than 24 h following collection. This is an inherent problem with the current referral pathway to a centralized testing laboratory where biopsy specimens are initially transferred to a local microbiology laboratory and then referred to the GBRU for culture and susceptibility testing. In this study, less than a quarter of gastric biopsies were received by the GBRU within 24 h from the date of collection. The implementation of a molecular testing workflow may prove useful in this instance for the detection of *H. pylori* and associated AMR in these specimens. However, commercial molecular assays currently available are only for the detection of known resistance mechanisms for clarithromycin (e.g. Allplex™ *H. pylori* and ClariR Assay, Seegene) or clarithromycin and levofloxacin (e.g. GenoType HelicoDR, Brucker), but their use in this setting could still have potential to improve overall patient management [9].

We found rates of resistance to metronidazole and clarithromycin remained high across the 5-year period, which may be due to these cultures of *H. pylori* having been recovered primarily from gastric biopsies taken from patients who would have already failed first- or second-line treatment regimes. Over 95.0% of cultures recovered from gastric biopsies exhibited reduced susceptibility to metronidazole; therefore, continuing to routinely test such refractory *H. pylori* cultures for this agent appears to be of limited use in guiding tailored treatment regimes in the UK at this stage of infection. There was also an apparent increasing trend in the last 5 years in the proportion of cultures exhibiting reduced susceptibility to levofloxacin from 24.0% in 2020 to 31.6% in 2024. However, since information regarding patient treatment is unknown, this observation would require further investigation to confirm. Nevertheless, there is a clear need to conduct a prospective point prevalence study in treatment-naïve patients of primary metronidazole, clarithromycin and levofloxacin resistance in *H. pylori* in England with an aim to inform national guidelines for the management of *H. pylori* infections.

## Conclusion

Antimicrobial resistance in *H. pylori* is a major contributing factor in failure to eradicate infections. Within the UK, there is a high prevalence of resistance to metronidazole and clarithromycin and an increasing resistance to levofloxacin in treatment refractory *H. pylori* cultures recovered from gastric biopsy material. There is an urgent need to establish UK primary resistance rates in treatment-naïve patients to support a review of regional first-line treatment regimes. The results in this study also support a need for rapid, local isolation of *H. pylori* by clinical microbiology laboratories, to maximize patient benefit due to the delays in sending gastric biopsies to the GBRU for culture having a negative effect on the probability of successfully recovering a viable strain for antimicrobial susceptibility testing. The implementation of molecular tests to accurately and rapidly detect antimicrobial resistance mechanisms in *H. pylori* may improve overall patient management.

## Abbreviations

AMR: antimicrobial susceptibility and resistance
COVID-19: coronavirus disease 2019
EUCAST: European Committee on Antimicrobial Susceptibility Testing
GBRU: Gastrointestinal Bacteria Reference Unit
UKHSA: UK Health Security Agency
